# Engineering Cytoplasmic Signaling of CD28ζ CARs for Improved Therapeutic Functions

**DOI:** 10.3389/fimmu.2020.01046

**Published:** 2020-06-19

**Authors:** Xianhui Meng, Ruirui Jing, Liling Qian, Chun Zhou, Jie Sun

**Affiliations:** ^1^Bone Marrow Transplantation Center of the First Affiliated Hospital, Zhejiang University School of Medicine, Hangzhou, China; ^2^Department of Cell Biology, School of Basic Medical Sciences, Zhejiang University, Hangzhou, China; ^3^Institute of Hematology, Zhejiang University & Laboratory of Stem Cell and Immunotherapy Engineering, Hangzhou, China; ^4^School of Public Health and Sir Run Run Shaw Hospital, Zhejiang University School of Medicine, Hangzhou, China

**Keywords:** chimeric antigen receptor, CD3ζ, ITAM, signal strength, cytotoxicity, persistence

## Abstract

Chimeric antigen receptor modified T cells (CAR-T) have yielded impressive clinical outcomes in treating hematopoietic malignancies. However, relapses have occurred in a substantial number of patients and limited the development of CAR-T therapy. Most underlying reasons for these relapses can be attributed to poor persistence and rapid exhaustion of CAR-T cells *in vivo*. Despite multiple strategies having been developed, how to improve CAR-T persistence or resist exhaustion while maintaining sufficient cytotoxic functions is still a great challenge. Here we discuss engineering cytoplasmic signaling as an important strategy for CAR optimization. This review summarizes recent advances showing that the anti-tumor function of CAR-T cells can be improved by optimizing the CD3ζ domain or downstream signaling of CD28ζ CAR.

## Introduction

Chimeric antigen receptor modified T cells (CAR-T) therapy achieved great success against hematological malignancies. Through genetic engineering with a chimeric antigen receptor (CAR), the modified T cells can be activated by specific antigens on the surface of target cancer cells and produce anti-tumor toxicity. As the recognition of antigens or activation of downstream signals does not depend on MHC molecules, CAR-T cells are anticipated to overcome the immune escape route of cancer cells through their down-regulated expression of MHC molecules (1). CAR-T therapy achieved a milestone in 2017 when two CD19 targeted CAR-T therapies (Yescarta and Kymriah) were approved by the US Food and Drug Administration (FDA). Nowadays, many emerging preclinical and clinical studies on multiple malignancies are demonstrating the significant anti-tumor potential of CAR-T therapies (2, 3).

Despite impressive clinical outcomes, there are still limitations of CAR-T therapies. Previous clinical studies indicated that although a large proportion of patients could achieve a complete remission (CR), some of them suffered disease relapse. For relapsed/refractory B-cell acute lymphoblastic leukemia (ALL) patients, although CR rate after initial anti-CD19 CAR-T therapy is higher than 70%, ~30 to 50% of these CR patients suffered disease relapse within 1 year of treatment (4). Among many strategies used to solve the problem of relapse, improving the persistence and attenuating exhaustion of CAR-T cells is regarded as a critical requirement for long-term tumor remission (5).

Strategies to optimize each module, especially the costimulatory domain of CARs, have been extensively reviewed elsewhere (6, 7). In this review, we will discuss recent novel strategies of CAR optimization. Particularly we will focus on the optimization of CD3ζ domain of CD28ζ CAR and its downstream signaling in an effort to improve CAR-T cell functions.

## Development of Different CAR Designs

As a CAR is designed to activate T cells in response to specific antigens, the essential CAR structure is the extracellular antigen recognition domain and the intracellular signal transduction domain. In the late 1980s, Eshhar et al. designed the first-generation CARs, which included an antibody derived single-chain variable fragment (scFv) as the antigen recognition domain, and a Fc receptor γ chain (FcRγ) or CD3ζ derived signal transduction domain (8). T cells equipped with first-generation CARs were demonstrated to recognize antigens in an MHC independent manner. However, these T cells only exhibited a weak anti-tumor activity and tended to be anergized or exhausted *in vivo* (9). The reason was attributed to the lack of costimulatory signals, which are the essential “second signal” to fully activate T cells (10). The second-generation CARs solved this problem by including an intracellular domain derived from costimulatory molecules such as CD28, ICOS, 4-1BB, OX40, or CD27. The incorporation of a costimulatory domain was a breakthrough for CAR-T therapy as it equipped CAR-T cells with potent *in vivo* anti-tumor activity (11–13). Subsequent studies have engaged in optimizing costimulatory domains for enhanced T cells activation. These studies led to the development of third-generation CARs (containing multiple costimulatory domains). In some preclinical studies, the incorporation of multiple costimulatory components has been demonstrated with improved anti-tumor functions. However, their therapeutic outcomes in recent clinical trials have shown modest benefits compared to second-generation CARs (14).

## Anti-Tumor Functions of CD28ζ and 4-1BBζ CARs

Until now, the most successful application of CAR-T therapy has been CD19-targeted CARs toward B cell malignancies such as non-Hodgkin lymphoma (NHL), chronic lymphocytic leukemia (CLL), and ALL (15). Despite different CAR designs, manufacturing, and clinical regimens, accumulated clinical trials have shown that CD19 CARs achieved 70–90% CR rates among children and adults with relapsed/refractory B-cell ALL (16).

In these clinical studies, most CARs contain either a CD28 or 4-1BB cytoplasmic domain as the costimulatory element. In the treatment of ALL, both CD28ζ and 4-1BBζ CARs achieved similar outcomes. However, in CLL and NHL, clinical trials indicated the superior efficacy of 4-1BBζ CAR compared to that of CD28ζ CAR (17). In a recent clinical study of NHL, a parallel comparison showed that both CD28ζ and 4-1BBζ CAR-T cells displayed similar anti-tumor efficacies within 3 months. However, CD28ζ, but not 4-1BBζ, CAR-T cells induced severe cytokine release syndrome (CRS) and neurotoxicity (18). These distinct clinical outcomes may be attributed to the different downstream signaling cascades invoked by the CD28 or 4-1BB cytoplasmic domain.

Endogenous CD28, as a member of the CD28 family, is known to induce signals via PI3K, NF-κB, Akt, Erk, and NFAT to regulate expression of T-bet, Eomes, and GATA3 (19). 4-1BB, as a member of the tumor necrosis factor receptor super family (TNFRSF), is known to activate downstream signals through the recruitment of TRAF proteins. Corresponding to their different signaling and regulation patterns, *in vitro* functional assays showed that CD28ζ CAR induced higher levels of released cytokines such as IL-2, IFNγ, and TNFα, and an enhanced cytotoxic effect than 4-1BBζ CAR (20). Kinetics and protein phosphorylation profile studies showed that CD28ζ CAR was more prone to activate effector T cell-associated genes, while 4-1BBζ CARs preferentially activated memory T cell-associated genes (21). In accordance with these findings, *in vivo* 4-1BBζ CAR was shown to promote the differentiation of central memory T cells, while CD28ζ CAR was more prone to promote the differentiation of effector memory T cells (17). As a result, 4-1BBζ CAR-T cells have been shown to have a superior *in vivo* persistence than CD28ζ CAR-T cells. As reported, the persistence of CD28ζ CAR-T cells is about 30 days, while 4-1BBζ CAR-T cells may exceed 4 years in some patients (17). The long persistence of 4-1BBζ CAR may be responsible for its comparable clinical efficacy with CD28ζ CAR in ALL despite its weaker cytotoxic effect (15). Collectively, the clinical outcomes of CD28- or 4-1BB-based CAR-T therapies suggest that cytotoxic effects and persistence properties are crucial factors affecting CAR-T therapeutic functions.

## Balancing Effector and Memory Differentiation Determining CAR-T Cell Functions

To understand the functions of engineered CAR-T cells, we may first review what happens to un-engineered native T cells. During T cell response to infection, recognition of a specific antigen induces naïve T cells activation, leading them into rapid proliferation and differentiation. This response will create a highly diverse T cell pool, in which the cooperation of effector and memory T cell subpopulations is indispensable for efficient antigen clearance (22). On one hand, the cytotoxic effect is mainly performed by effector T cell subsets. By producing cytokines and cytotoxic molecules, effector T cells can directly kill target cells. On the other hand, following antigen clearance most activated T cells die while a small pool of memory T cells can persist for a rapid response to the antigen re-challenge (23, 24). Particularly, in response to cancers or chronic infection, T cells have to deal with persistent antigen stimulation. In that circumstance, T cells may fail to differentiate into a memory subset and become exhausted. The exhausted T cells lose effector functions and are unable to efficiently clear target cells (22).

The response of CAR-T cells to tumors could follow similar processes. For rapid and efficient tumor clearance, CAR-T cells need to perform effective cytotoxicity, which rely on the effector T cell subset. However, overpowering cytotoxicity can induce certain issues and impair therapeutic functions. One issue is the severe side effects including CRS, which is characterized by massive synchronized T cell activation and the release of large amounts of cytokines, and immune effector cell-associated neurotoxicity syndrome. Even though macrophages have been identified as a major source for CRS (25, 26), CAR designs that reduce effector functions of CAR-T cells have been shown to decrease CRS in patients (27). Another issue is activation-induced cell death (AICD) and exhaustion of T cells. The enhanced differentiation toward effector T cell subsets inevitably attenuates the development of a memory T cell pool (28–30). The weak memory T cell subset attenuates T cell persistence and is responsible for tumor relapse. Therefore, balancing the effector and memory function of CAR-T cells is critical for effective relapse-free anti-tumor efficacy.

As the weak persistence of CD28ζ CAR-T cells is an important reason for unfavorable clinical outcomes, improving the persistence of CD28ζ CAR-T cells while keeping their potent effector functions is a good strategy to improve their therapeutic functions. For this purpose, the signal transduction of CD28ζ CAR can be properly modified to balance T cell differentiation in response to antigen stimulation.

## TCR and CD28-Based Signal Transduction

**Figure 1:**
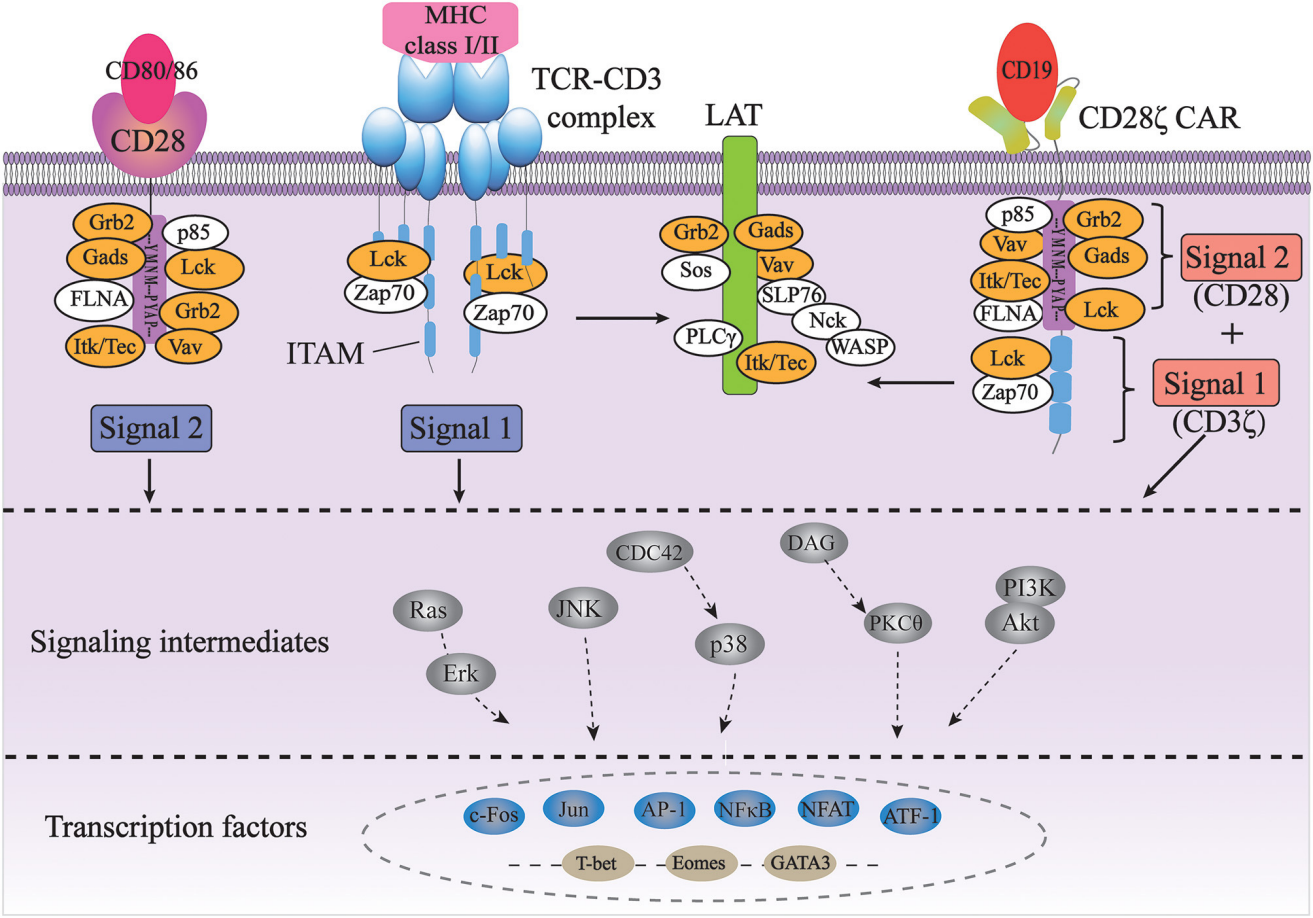
Signal transduction of TCR and CAR for T cell activation. The full activation of T cells requires TCR signaling through the CD3 complex (signal 1) and costimulatory CD28 signaling (signal 2); CD28ζ CAR integrates with the CD3ζ and CD28 domains, transducing two signals together in an antigen-dependent manner (orange color indicates common players shared by TCR/CD3 and CD28 pathways).

Currently, the precise molecular mechanism of CAR-induced T cell activation is still not well-understood. As the signal transduction of CARs largely depends on the signaling domains of the original immunoreceptors, it is assumed that the CARs transduce intracellular signals similar to endogenous TCRs and costimulatory molecules (Figure 1).

The TCR complex consists of a TCRαβ or TCRγδ heterodimers and a CD3 complex containing the CD3γε, CD3δε, and CD3ζζ dimers. While TCRαβ (or TCRγδ) subunits recognize antigens through their specific extracellular regions, the CD3 complex mainly carries out signal transduction functions in the complex through its well-conserved immunoreceptor tyrosine-based activation motifs (ITAMs) (31). First identified based on their sequence homology, ITAMs consist of two consecutive YxxL/I motifs separated by a defined number of amino acids (YxxL/I-X_6−8_-YxxL/I) (32). ITAMs are usually found in receptors expressed in hematopoietic cells and are especially well studied in the context of TCR signaling. The CD3γ, CD3δ, and CD3ε chains each contain one ITAM, while the CD3ζ chain contains three ITAMs. TCR binding to peptide-MHC leads to the activation of a Src family kinase Lck, which phosphorylates two tyrosine residues in each of the ITAMs in CD3 (33). Each bisphosphorylated ITAM then gains the ability to bind to the two tandem SH2 domains of a Syk family kinase, ZAP-70. This interaction brings ZAP-70 in close proximity to Lck, resulting in the phosphorylation and activation of ZAP-70 by Lck. Activated ZAP-70 further phosphorylates its downstream targets, such as adaptor protein LAT and SLP-76. Phosphorylated LAT and SLP-76 provide scaffolds for many other proteins, such as PLC-γ, Grb2/Sos, Gads and Itk, Vav, and Nck, eventually leading to calcium mobilization, Ras/Erk activation, actin cytoskeletal rearrangement, and ultimately activation of gene expression (31). Therefore, the ITAMs in CD3 are the major if not the only signaling moieties in TCR signaling.

Besides TCR signaling (Signal 1), full activation and expansion of T cells also requires signaling through costimulatory receptors such as CD28 (Signal 2). Interaction of CD3 with CD28 has been demonstrated to play a crucial role in modifying the endogenous TCR signal (34, 35). CD28, on the other hand, doesn't contain any ITAM. Instead, its cytoplasmic domain contains a YMNM motif that gets phosphorylated upon CD28 binding to its ligand CD80/CD86, which can bind to the p85 subunit of PI3K and Grb2/Gads. Additionally, proline-rich regions of CD28 can interact with Itk, Tec, Lck, Grb2/Vav, and FLNA (36, 37). Therefore, antigen-binding initiated TCR signaling through CD3 and CD80/86-binding initiated CD28 signaling share many common players, such as Grb2, Vav, Gads, Lck, and Itk. In addition, the activation of both pathways occurs in the signaling complexes assembled near the plasma membrane at the immunological synapse, physically bringing signaling molecules from two pathways together in space (38, 39). Last but not least, CD28-induced calcium signaling occurs seconds after TCR-initiated intracellular calcium increase, if not sooner, suggesting the temporal proximity/closeness of the two pathways (40). All of these suggest a synergetic spatiotemporal collaboration between TCR-CD3 and CD28 signaling, which contributes to the highly ordered signal transduction of T cells.

## Stoichiometry Imbalance of CD3ζ and CD28 Signaling in CD28ζ CAR

In CD28ζ CAR, CD28 and CD3ζ domain are fused together to implement signal transduction. Therefore, it is assumed that the synergetic effect of CD3 and CD28 signaling also plays a crucial role in regulating downstream signaling and affecting T cell function. Nevertheless, when comparing CD28ζ CAR signaling to TCR signaling, some major differences are obvious due to the fusion of two cytosolic domains. First is that in CAR, CD28 signaling domain is *in cis* with CD3ζ signaling domain, while endogenous CD28 is recruited into the immune synapse and co-localized with CD3ζ *in trans*. Second, CD28 activation is concurrent with CD3ζ activation in CAR while CD28 costimulation occurs seconds after TCR ligation. Third, in human T cells, CD28 and TCR are normally expressed at ~6 × 10^4^ and ~2 × 10^4^ molecules per cell (41). Therefore, three molecules of CD28 can provide signaling support for one molecule of TCR/CD3. However, second-generation CD28ζ CAR has the design of fused CD28 and CD3ζ cytosolic domains, fixing the stoichiometry ratio of them to be 1:1. The redundancy of CD3ζ signaling may disturb the signaling homeostasis of CAR and result in improper enhancing of T cell stimulation, which accounts for the poor persistence of CAR-T cells. Balancing the costimulation signaling and activation signaling may help solve the existing overstimulation problem of CD28ζ CAR.

## Fine-Tuning ITAM Numbers and Positions of CD3ζ Domain in CD28ζ CAR

Although the detailed mechanisms regulating the formation of effector and memory T cell pools are still elusive, it is generally considered that signal strength is an important determinant for T cell fate (42). In native T cells, the TCR complex binds to antigens and transduces the binding across the plasma membrane to intracellular signals. It has been reported that weak TCR signals favor memory T cells differentiation, whereas strong TCR signals promote the formation of effector T cell subsets (43, 44).

Multiple ITAMs in CD3 and TCR complexes have been proposed to amplify TCR signals (31, 45). So, the number and type of signaling domains matter in TCR signal strength. In animal models, mice with fewer than seven CD3 ITAMs developed a lethal multiorgan autoimmune disease (46). Further analysis demonstrated that the efficiency of Notch1 induced c-Myc expression was reduced in T cells expressing CD3 with two or four ITAMs, which resulted in impaired cellular proliferation (47). These studies suggested a linear relationship between the number of ITAMs and the proliferative ability of naïve T cells. The type of ITAMs also matters. The three ITAMs in CD3ζ differ in their primary amino acid sequences as well as their positions relative to the plasma membrane (namely ITAM1, ITAM2, and ITAM3 from membrane proximal to distal), therefore their ability of being phosphorylated by Lck or binding to ZAP70 upon phosphorylation are different (48). Mutations of ITAM1 and ITAM2 in CD3ζ significantly impaired signal transduction and induced cell death. However, mutation of ITAM3 in CD3ζ did not induce cell death but rather increased IL-2 secretion and MAPK phosphorylation (49). Therefore, ITAMs in CD3ζ are functionally different in regulating T cell activation.

In CAR-T cells, the CAR molecules are responsible for antigen recognition and signal transduction. Therefore, it is logical to modulate T cell differentiation potentials by controlling the signal strength of CARs. Nowadays, many strategies have been developed to optimize CAR designs. Modification of the extracellular scFv, the hinge, the transmembrane domain, and the costimulatory domains of CAR have been evaluated by multiple studies (6). However, limited works have focused on the modification of the CD3ζ domain of CAR (50, 51).

Actually, the first-generation CAR was designed to have either a CD3ζ chain or a FcRγ as the intracellular signaling component. T cells with first-generation CD3ζ CAR were later demonstrated to have a greater cytotoxicity and anti-tumor functions than those with FcRγ CAR (52). And the greater cytotoxicity was attributed to the presence of three ITAMs in CD3ζ compared to the one ITAM in FcRγ. Subsequently, the CD3ζ instead of FcRγ chain was preferentially used in the next generation CAR designs. However, in second-generation CD28ζ CARs, incorporation of CD28 costimulatory domains provides a quantitative support for downstream signaling and the signal transduction of CD3ζ domain may be significantly affected by the synergetic effect with CD28 signaling (41). Therefore, further evaluation may be required to ascertain the suitability of CD3ζ with three ITAMs in the context of CD28ζ second-generation CAR.

Meanwhile, several *in vitro* studies have indicated the significant effect of CD3ζ modification on CAR functions. A study of CD28-based ErbB2 CAR with only one ITAM at second position showed reduced apoptosis upon T cell activation *in vitro* (53). Increasing the number of ITAMs from three to six was shown to increase the efficiency of T cell activation (50). Interestingly, decreasing the ITAM number from three to two showed equivalent T cell activation for target cells expressing CD19 with high density (50). Although limited to *in vitro* evaluations, these studies informed the importance of ITAMs on CAR-T cell functions.

In a recent study, Feucht and colleagues designed CD19-targeted CD28ζ CAR (1928ζ CAR)-based new CARs with a defined ITAM number and position to see whether these CARs could overcome some of the adverse issues (54). The study first showed that CAR with only ITAM1 (referred as 1XX CAR) or only ITAM2 (referred as X2X CAR) induced comparable *in vitro* cytotoxicity with the original CD28ζ CAR, while CAR with only ITAM3 (referred as XX3 CAR) led to impaired cytotoxic function (54). In an *in vivo* animal model, the study further showed that 1XX CAR achieved durable and complete tumor remission. However, X2X CAR and XX3 CAR both failed to achieve complete tumor remission. More importantly, 1XX CAR treatment significantly increased the mice survival rate even better than the original CD28ζ CAR (54).

The improved therapeutic function of 1XX CAR can be attributed to the increased persistence *in vivo*. Firstly, in 1XX CAR treated mice, there was a higher accumulation of CAR-T cells at the tumor site, and both CD4^+^ and CD8^+^ CAR-T cell subsets showed a higher percentage of CD62L^+^CD45RA^−^ central memory T cells (T_CM_) and a lower fraction of terminally differentiated CD62L^−^CD45RA^+^ effector cells (T_EFF_) (54). Secondly, results from antigen re-exposure assay and exhaustion markers detection collectively demonstrated that exhaustion was rapidly acquired in CD28ζ CAR-T cells but largely attenuated in 1XX CAR-T cells. Thirdly, following tumor rechallenge, 1XX CAR achieved complete tumor control, while CD28ζ CAR failed to control tumor rechallenge, correlating the long persistence with low relapse (54).

Finally, at transcriptional level, the study revealed that wild type CD28ζ CAR-T cells were similar to T_EFF_ cells with the highest expression of effector differentiation related genes such as T-bet, PRDM1, and ID-2. XX3 CAR-T cells, on the other hand, were more similar to naïve T cells with the most significant down-regulation of T cell differentiation related genes and up-regulation of naïve/memory-associated genes such as TCF7, BCL6, LEF1, and KLF2. However, 1XX CAR-T cells exhibited a greater similarity to stem cell memory T cells (T_SCM_) with a more balanced expression of differentiation and memory related genes (54).

**Figure 2:**
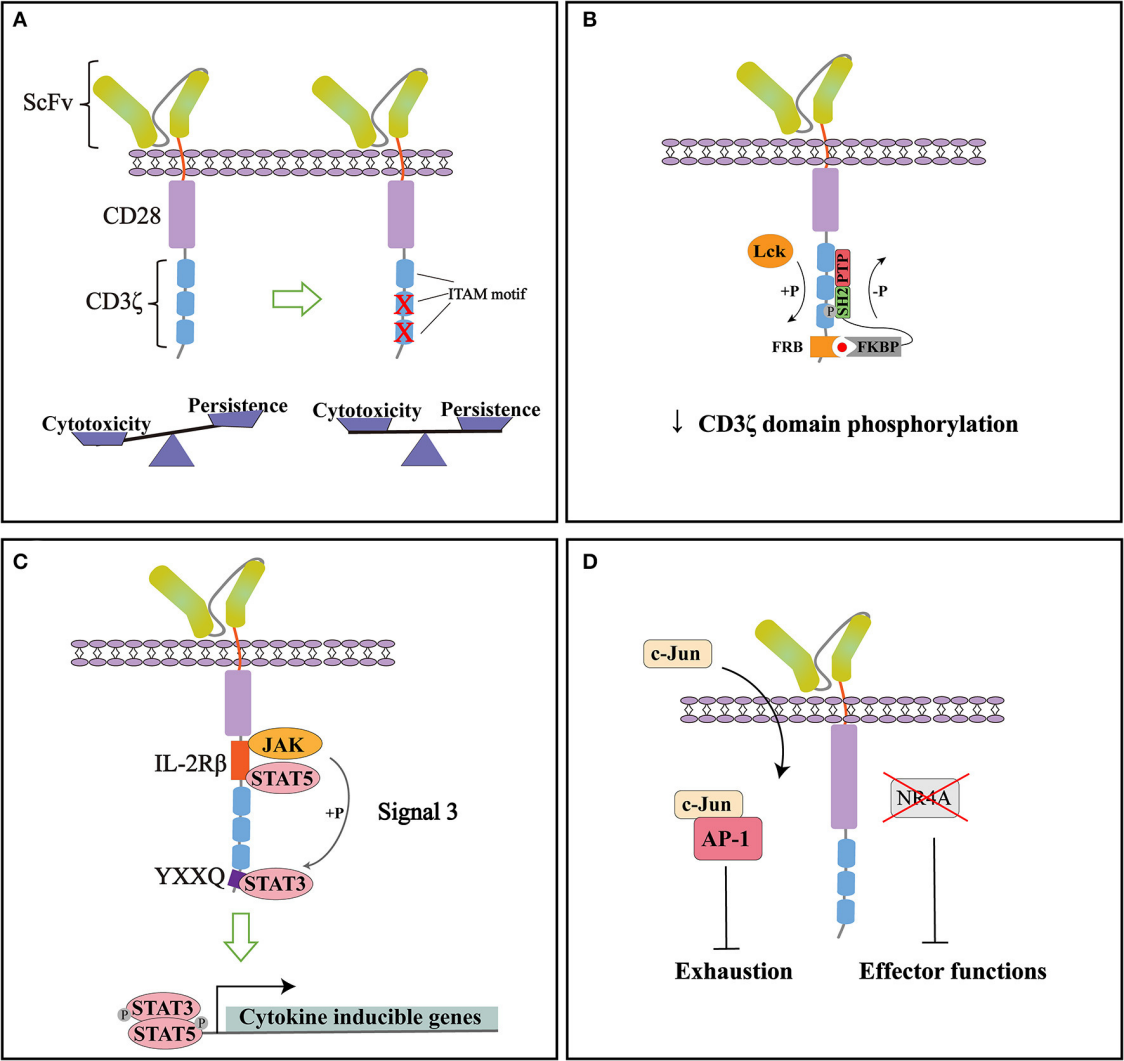
Representative strategies to optimize cytoplasmic signaling of CD28ζ CAR. **(A)** By mutating the two membrane-distal ITAMs while keeping the membrane-proximal ITAM intact (1XX), the cytotoxicity and persistence of CARs can be balanced to improve therapeutic functions. **(B)** Small molecule-induced SHP1 phosphatase binding can tune down the basal phosphorylation level of CD28ζ CAR and reduce its antigen-dependent response. **(C)** Incorporation of signaling modules from cytokine receptor (signal 3) specifically activates the JAK-STAT pathway and improves the persistence and anti-tumor effect of CD28ζ CAR. **(D)** CAR signaling can be optimized through direct manipulation of transcription factors. NR4A knockout or c-Jun over-expression can counteract the exhaustion of CAR-T cells and improve their anti-tumor efficacy.

Taken together, CAR modified with 1XX achieved a superior anti-tumor efficacy, making it a great candidate for next step clinical trials (Figure 2A). By modifying the ITAM configuration of CD3ζ domain, the persistence of CAR-T cells can be improved while keeping the desired cytotoxic effect.

## Modifying Downstream Signaling of CD28ζ CAR

With improved understanding of CAR signaling mechanisms, additional strategies can be used to modify downstream signaling of CD28ζ CAR. A recent study by Sun and colleagues found that CD28ζ CAR had higher basal phosphorylation of CD3ζ domain and higher antigen-dependent T cell activation than 4-1BBζ CAR. To tune down its phosphorylation state, they introduced an FRB element into the intracellular domain of CD28ζ CAR and designed a fusion protein linking FKBP and SHP1 phosphatase. The administration of small molecule AP21967 will induce the heterodimerization of FKBP with FRB, recruiting SHP1 to the CD3ζ domain and promote its dephosphorylation (Figure 2B). In a humanized mouse model, they demonstrated that this design effectively suppressed tumor growth without significant weight loss of the mice. The reduced cytokine release in the plasma after AP21967 administration indicated that toxicities such as CRS could be ameliorated by this strategy (55). Therefore, the cytotoxicity of CAR-T cells can be precisely controlled by small molecules to prevent possible severe side effects.

Cytokine signaling is generally considered important for optimal T cell activation as signal 3. Kagoya et al. showed that adding signaling modules from cytokine receptors can also be beneficial to CD28ζ CAR function. They inserted an IL-2Rβ domain between CD28 and CD3ζ, and a YXXQ motif at the distal region of CD3ζ domain (Figure 2C). The engineered CAR recruited JAK, STAT3, and STAT5 to activate the JAK-STAT pathway upon antigen stimulation. Gene expression analysis showed that the incorporation of these modules not only preferentially activated IL21-induced genes and STAT3 targets, but enriched genes associated with cytolytic activity. Compared to the original CD28ζ CAR, the engineered version achieved a greater proliferation ability and maintained more memory T cells *in vitro* even after repeated stimulation. More importantly, the engineered CAR showed superior anti-tumor functions in multiple mouse models, with a high percentage of CD8^+^CAR-T cells in peripheral blood, significantly reduced tumor growth, and the prolonged overall survival of mice. These data suggested that combining signals from cytokines can enhance persistence and promote the anti-tumor effect of CAR (56).

Moreover, activation and exhaustion of CAR-T cells can be balanced by targeting transcription factors. In a recent study, Chen et al. revealed that NR4A1, NR4A2, and NR4A3 are the key transcription factors that drive T cell dysfunction. NR4A triple knockout down-regulated the expression of PD-1 and TIM3. CAR-T cells lacking three NR4A proteins showed an enhanced effector function and anti-tumor effect (57). On the other hand, Lyn and colleagues developed a CAR-T exhaustion model and identified that the abnormal expression of JunB, BATF, and IRF4 disrupted the functions of AP-1 and are responsible for T cell exhaustion. Further genetic analysis revealed that in exhausted T cells, AP-1 was prone to interact with JunB, BATF3, IRF4, etc. This interaction antagonized the binding of AP-1 with its canonical factor c-Jun and resulted in exhaustion gene expression. To counteract this effect, they overexpressed c-Jun in CAR-T cells, which significantly promoted IL-2 and IFNγ expression, increased the frequency of memory T cells subsets, and improved tumor-free survival of mice. Therefore, c-Jun overexpression could be an effective method to rescue exhausted CAR-T cells and enhance their anti-tumor functions (Figure 2D) (58).

## Conclusion

Although CAR-T therapy has shown impressive potential in the treatment of previously uncurable malignancies, some barriers still need to be overcome. Among them, the high rate of tumor relapse is a critical concern. To accomplish complete tumor elimination without relapse, CAR-T cells need to persist with sufficient cytotoxicity and limited exhaustion. This partly relies on the fine-tuning modification of CAR signaling. Herein, we have discussed strategies to optimize the CD3ζ domain or downstream signaling of CARs for improved anti-tumor efficacy. CD3ζ chain has been widely used in CAR designs since the first-generation CARs. However, very few studies have evaluated its suitability in second-generation CARs. This review highlighted CD3ζ domain modification as one important strategy to optimize CAR functions. Particularly, we provided evidence showing that the cytotoxicity and persistence of CAR-T cells can be balanced by modifying ITAM motifs of the CD3ζ domain. Moreover, recent advances in CAR signaling provide exciting new strategies to optimize CAR function. These studies highlighted that modifying cytoplasmic signaling of CAR is effective in improving CAR-T efficacy. This review has focused on the optimization of CD28-based CAR. As the signaling pathway of 4-1BB is significantly different from CD28, it will be interesting to see whether these strategies are applicable to 4-1BBζ CAR. Further studies are encouraged to investigate whether modifying ITAM configurations or downstream signaling contributes to improved functions of other antigen-targeted and costimulatory domain-based CARs.

## Author Contributions

XM, RJ, and LQ collected data and wrote the manuscript. JS and CZ wrote the manuscript and supervised the research. All authors read and approved the final manuscript.

## Conflict of Interest

The authors declare that the research was conducted in the absence of any commercial or financial relationships that could be construed as a potential conflict of interest.

## Abbreviations

CAR: chimeric antigen receptor
CAR-T: chimeric antigen receptor modified T cells
FDA: Food and Drug Administration
CR: complete remission
scFv: single-chain variable fragment
FcRγ: Fc receptor γ chain
NHL: non-Hodgkin lymphoma
CLL: chronic lymphocytic leukemia
ALL: acute lymphoblastic leukemia
CRS: cytokine release syndrome
AICD: activation-induced cell death
ITAM: immunoreceptor tyrosine-based activation motifs
T_CM_: central memory T cells
T_EFF_: effector cells
T_SCM_: stem cell memory T cells.
